# 4.F. Skills building seminar: How can we achieve effective interdisciplinarity in digital public health practice?

**DOI:** 10.1093/eurpub/ckac129.228

**Published:** 2022-10-25

**Authors:** Organised by: Leibniz ScienceCampus Digital Public Health (Germany), Chair persons: Laura Maass (Germany), Chen-Chia Pan (Germany)

## Abstract

Digital technology, which is driven by technical opportunities rather than by demands, can fail to realise the critical objectives of evidence-based public health and pose novel ethical, legal, sociocultural, and equity-related challenges. While individual health already needs various disciplines to be adequately addressed, even more fields gain importance when it comes to population health. Public health requires collaboration across all domains: Those that tackle an individual's health but also those that set the framework for health practices (e.g., law, medicine, psychology, ethics, or sociology), measure health outcomes on a population-level (e.g., epidemiology), or that develop interventions to improve the populations’ health (e.g., health promotion, health management, implementation science). During the last years, health has become more and more digitalised. Adding the digital level to public health also brings more and increasingly important players on the stage (e.g., human-computer-interaction, computer science, or engineering), who are needed to develop and improve evidence-based digital technologies and innovations on a population level. Having various disciplines collaborating with each other can offer new opportunities but also create new challenges if there is no mutual understanding of digital public health. This skill-building workshop aims to foster awareness of interdisciplinary approaches in public health research and practice. Two short introductory presentations (10 minutes each) will form the frame for the workshop. The first talk will introduce participants to the idea of interdisciplinarity in research and practice and highlight the prerequisites for effective and sustainable interdisciplinary collaborations. The second talk will build on this understanding and present a holistic (digital) public health model that displays how different disciplines contribute to the field and how an interdisciplinary approach can be used to overcome individual challenges. Based on the talks, participants will be invited to work on a case study regarding mobile mental health apps as a digital public health intervention. We will apply the world coffee method, in which each table will display one individual discipline (epidemiology, health economics, psychology, law, ethics, and computer science). An expert in the field will support each table to assist the participants if needed. Every participant will be able to discuss their ideas at three tables (10 min. each). The goal will be to work out how one could best integrate the individual disciplines when developing, implementing, and evaluating mental health apps in the digital public health environment. We discuss what each discipline can contribute to the topic and for which aspects it needs support from other scientific fields. The final results will be collected and summarised as a chapter in a digital public health handbook published by the Leibniz ScienceCampus Digital Public Health.

**Key messages:**

• Considering different disciplines when developing and implementing digital public health interventions improves the interventions’ outcomes and long-term acceptability in user groups.

• Interdisciplinarity in digital public health requires a mindset from researchers that is characterised by empathy/curiosity towards other disciplines and a common understanding of the intervention.

